# Association between Prenatal Exposure to Antiretroviral Therapy and Birth Defects: An Analysis of the French Perinatal Cohort Study (ANRS CO1/CO11)

**DOI:** 10.1371/journal.pmed.1001635

**Published:** 2014-04-29

**Authors:** Jeanne Sibiude, Laurent Mandelbrot, Stéphane Blanche, Jérôme Le Chenadec, Naima Boullag-Bonnet, Albert Faye, Catherine Dollfus, Roland Tubiana, Damien Bonnet, Nathalie Lelong, Babak Khoshnood, Josiane Warszawski

**Affiliations:** 1 Hôpital Louis Mourier, Assistance Publique–Hôpitaux de Paris, Colombes, France; 2 Centre de Recherche en Épidémiologie et Santé des Populations, INSERM U1018, Le Kremlin-Bicêtre, France; 3 Université Paris Diderot—Paris 7, Paris, France; 4 Hôpital Necker, Assistance Publique–Hôpitaux de Paris, Paris, France; 5 EA 3620, Université Paris Descartes 5, Paris, France; 6 Institut National d'Etudes Démographiques, Paris, France; 7 Hôpital Robert Debré, Assistance Publique–Hôpitaux de Paris, Paris, France; 8 Hôpital Trousseau, Assistance Publique–Hôpitaux de Paris, Paris, France; 9 Hôpital Pitié Salpétrière, Assistance Publique–Hôpitaux de Paris, Paris, France; 10 INSERM U943, Paris, France; 11 Pediatric Cardiology, M3C Necker-Enfants Malades, Assistance Publique–Hôpitaux de Paris, Université Paris Descartes, Paris, France; 12 INSERM UMR S953, Université Paris 6, Paris, France; 13 Université Paris Sud, Le Kremlin-Bicêtre, France; Hospital Clinic Barcelona, Spain

## Abstract

**Background:**

Antiretroviral therapy (ART) has major benefits during pregnancy, both for maternal health and to prevent mother-to-child transmission of HIV. Safety issues, including teratogenic risk, need to be evaluated. We estimated the prevalence of birth defects in children born to HIV-infected women receiving ART during pregnancy, and assessed the independent association of birth defects with each antiretroviral (ARV) drug used.

**Methods and Findings:**

The French Perinatal Cohort prospectively enrolls HIV-infected women delivering in 90 centers throughout France. Children are followed by pediatricians until 2 y of age according to national guidelines.

We included 13,124 live births between 1994 and 2010, among which, 42% (*n = *5,388) were exposed to ART in the first trimester of pregnancy. Birth defects were studied using both European Surveillance of Congenital Anomalies (EUROCAT) and Metropolitan Atlanta Congenital Defects Program (MACDP) classifications; associations with ART were evaluated using univariate and multivariate logistic regressions. Correction for multiple comparisons was not performed because the analyses were based on hypotheses emanating from previous findings in the literature and the robustness of the findings of the current study. The prevalence of birth defects was 4.4% (95% CI 4.0%–4.7%), according to the EUROCAT classification. In multivariate analysis adjusting for other ARV drugs, maternal age, geographical origin, intravenous drug use, and type of maternity center, a significant association was found between exposure to zidovudine in the first trimester and congenital heart defects: 2.3% (74/3,267), adjusted odds ratio (AOR) = 2.2 (95% CI 1.3–3.7), *p = *0.003, absolute risk difference attributed to zidovudine +1.2% (95% CI +0.5; +1.9%). Didanosine and indinavir were associated with head and neck defects, respectively: 0.5%, AOR = 3.4 (95% CI 1.1–10.4), *p = *0.04; 0.9%, AOR = 3.8 (95% CI 1.1–13.8), *p = *0.04. We found a significant association between efavirenz and neurological defects (*n = *4) using the MACDP classification: AOR = 3.0 (95% CI 1.1–8.5), *p = *0.04, absolute risk +0.7% (95% CI +0.07%; +1.3%). But the association was not significant using the less inclusive EUROCAT classification: AOR = 2.1 (95% CI 0.7–5.9), *p = *0.16. No association was found between birth defects and lopinavir or ritonavir with a power >85% for an odds ratio of 1.5, nor for nevirapine, tenofovir, stavudine, or abacavir with a power >70%. Limitations of the present study were the absence of data on termination of pregnancy, stillbirths, tobacco and alcohol intake, and concomitant medication.

**Conclusions:**

We found a specific association between in utero exposure to zidovudine and heart defects; the mechanisms need to be elucidated. The association between efavirenz and neurological defects must be interpreted with caution. For the other drugs not associated with birth defects, the results were reassuring. Finally, whatever the impact that some ARV drugs may have on birth defects, it is surpassed by the major role of ART in the successful prevention of mother-to-child transmission of HIV.

*Please see later in the article for the Editors' Summary*

## Abstract 2

Jeanne Sibiude and colleagues use the French Perinatal Cohort to estimate the prevalence of birth defects in children born to HIV-infected women receiving antiretroviral therapy during pregnancy.

*Please see later in the article for the Editors' Summary*

## Editors' Summary

**Background:**

AIDS and HIV infection are commonly treated with antiretroviral therapy (ART), a combination of individual drugs that work together to prevent the replication of the virus and further spread of the infection. Starting in the 1990s, studies have shown that ART of HIV-infected women can substantially reduce transmission of the virus to the child during pregnancy and birth. Based on these results, ART was subsequently recommended for pregnant women. Since 2004, ART has been standard therapy for pregnant women with HIV/AIDS in high-income countries, and it is now recommended for all HIV-infected women worldwide. Several different antiviral drug combinations have been shown to be effective and are used to prevent mother-to-infant transmission. However, as with any other drugs taken during pregnancy, there is concern that ART can harm the developing fetus.

**Why Was This Study Done?:**

Several previous studies have assessed the risk that ART taken by a pregnant woman might pose to her developing fetus, but the results have been inconsistent. Animal studies suggested an elevated risk for some drugs but not others. While some clinical studies have reported increases in birth defects in children born to mothers on ART, others have shown no such increase.

The discrepancy may be due to differences between the populations included in the studies and the different methods used to diagnose birth defects. Additional large studies are therefore necessary to obtain more and better evidence on the potential harm of individual anti-HIV drugs to children exposed during pregnancy. So in this study, the authors conducted a large cohort study in France to assess the relationship between different antiretroviral drugs and specific birth defects.

**What Did the Researchers Do and Find?:**

The researchers used a large national health database known as the French Perinatal Cohort that contains information on HIV-infected mothers who delivered infants in 90 centers throughout France. Pediatricians follow all children, whatever their HIV status, to two years of age, and health statistics are collected according to national health-care guidelines. Analyzing the records, the researchers estimated the rate at which birth defects occurred in children exposed to antiretroviral drugs during pregnancy.

The researchers included 13,124 children who were born alive between 1994 and 2010 and had been exposed to ART during pregnancy. Children exposed in the first trimester of pregnancy, and those exposed during the second or third trimester, were compared to a control group (children not exposed to the drug during the whole pregnancy). Using two birth defect classification systems (EUROCAT and MACDP—MACDP collects more details on disease classification than EUROCAT), the researchers sought to detect a link between the occurrence of birth defects and exposure to individual antiretroviral drugs.

They found a small increase in the risk for heart defects in children with exposure to zidovudine. They also found an association between efavirenz exposure and a small increase in neurological defects, but only when using the MACDP classification system. The authors found no association between other antiretroviral drugs, including nevirapine (acting similar to efavirenz); tenofovir, stavudine, and abacavir (all three acting similar to zidovudine); and lopinavir and ritonavir (proteinase inhibitors) and any type of birth defect.

**What Do These Findings Mean?:**

These findings show that, overall, the risks of birth defects in children exposed to antiretroviral drugs in utero are small when considering the clear benefit of preventing mother-to-child transmission of HIV. However, where there are safe and effective alternatives, it might be appropriate to avoid use by pregnant women of those drugs that are associated with elevated risks of birth defects.

Worldwide, a large number of children are exposed to zidovudine in utero, and these results suggest (though cannot prove) that these children may be at a slightly higher risk of heart defects. Current World Health Organization (WHO) guidelines for the prevention of mother-to-child transmission no longer recommend zidovudine for first-line therapy.

The implications of the higher rate of neurological birth defects observed in infants exposed to efavirenz in the first trimester are less clear. The EUROCAT classification excludes minor neurological abnormalities without serious medical consequences, and so the WHO guidelines that stress the importance of careful clinical follow-up of children with exposure to efavirenz seem adequate, based on the findings of this study. The study is limited by the lack of data on the use of additional medication and alcohol and tobacco use, which could have a direct impact on fetal development, and by the absence of data on birth defects and antiretroviral drug exposure from low-income countries. However, the findings of this study overall are reassuring and suggest that apart from zidovudine and possibly efavirenz, other antiretroviral drugs are not associated with birth defects, and their use during pregnancy does not pose a risk to the infant.

**Additional Information:**

Please access these websites via the online version of this summary at http://dx.doi.org/10.1371/journal.pmed.1001635.

- This study is further discussed in a *PLOS Medicine* Perspective by Mofenson and Watts
- The World Health Organization has a webpage on mother-to-child transmission of HIV
- The US National Institutes of Health provides links to additional information on mother-to-child transmission of HIV
- The Elizabeth Glaser Pediatric AIDS Foundation also has a webpage on mother-to-child transmission
- The French Perinatal Cohort has a webpage describing the cohort and its main publications (in French, with a summary in English)

## Introduction

In France and other industrialized countries, the use of antiretroviral therapy (ART) during pregnancy has led to a spectacular decrease of the mother-to-child transmission rate of HIV-1, from about 20% down to about 1% currently [1]–[3]. Since 2004, standard care is to treat every HIV-infected pregnant woman with triple-combination ART [4]. Many HIV-infected women are already taking these drugs for their own health when conception occurs, or start during the first trimester. Concern has been raised about the potential toxicity of these drugs during fetal development [5]–[8].

The risk of birth defects associated with ART has been investigated following an animal study suggesting a teratogenic effect in monkeys exposed in utero to efavirenz [9]. Results from clinical studies were discrepant. A large meta-analysis found no increased risk of birth defects [10], despite the inclusion of two recent prospective studies that found an increased risk with first-trimester exposure to efavirenz [11],[12]. The largest database for birth defects and ART today is the Antiretroviral Pregnancy Registry (APR), with 15,451 births included [13]. To date, the only antiretrovirals (ARVs) for which use during the first trimester is associated with increased rates of overall birth defects in the APR compared with the general population (2.8%) are nelfinavir (3.9%) and didanosine (4.6%); no association with specific birth defects was identified for these two drugs. Two other studies found an association between congenital heart defects (CHDs) and ART: zidovudine in one of these studies (adjusted odds ratio [AOR] = 2.04 [95% CI 1.03–4.05] for zidovudine in the first trimester compared to no zidovudine in the first trimester) [11], and any kind of ART in the other (rate of CHDs = 2.5% for children exposed in the first trimester versus 0.8% for infants exposed only later in pregnancy, *p = *0.02) [14].

The diversity of the results may be due to differences between populations and the variety of follow-up protocols that determine how birth defects are diagnosed (Table 1). Moreover, definitions of birth defects differed between studies. The Metropolitan Atlanta Congenital Defects Program (MACDP) classification was most often used, and almost always adapted as described for the APR [13]. A recent Italian study found no association between ART and birth defects using the APR-modified MACDP classification [15]. Only one study used the European Surveillance of Congenital Anomalies (EUROCAT) classification, which is less inclusive than the MACDP classification [16], and several studies provided no information about the system of classification used [17]–[19]. Many studies suffered from lack of power. Some cases were enrolled in more than one study, leading to lower precision of risk estimates than if they were provided by totally independent studies [7],[8].

**Table 1:** Main studies on ART exposure and birth defects.

Study and Year	Time Period	ARV Drugs Compared	*N* (Exposed)	Percent with BD	Conclusion
**Knapp ** [12] ** 2012**	2002–2007	**All children**	1,112	5.5	Significant difference for EFV
		EFV, unexposed	1,055	5.2	
		EFV, 1st T	47	12.8	
		EFV, 2nd–3rd T	9	0	
**APR ** [13] ** 2013**	1989–2013	**All children**	15,451	2.9	No difference between 1st T and later exposure for any drug
		Any ART, 1st T	6,526	2.9	
		Any ART, 2nd–3rd T	8,523	2.8	
		Didanosine, 1st T	416	4.8	Didanosine and nelfinavir have higher rate of BDs than general population
		Nelfinavir, 1st T	1,211	3.9	
**Ford ** [10] ** 2011**	Until 2011	EFV, 1st T	1,290	2.9	No difference
		No EFV	8,122	3.9	
**Watts ** [14] ** 2011**	1997–2000	**All ARTchildren**	1,414	4.2	No difference for overall defects
		Any ART, 1st T	636	4.7	
		Any ART, 2nd–3rd T	778	3.9	
		**Heart defects**			Significantly more heart defects for exposure in the 1st T
		Any ART, 1st T		2.5	
		Any ART, 2nd–3rd T		0.8	
**Brogly ** [11] ** 2010**	1993–2000	**All children**	2,033	5.3	Significant difference for EFV
		Any ART, 1st T	763	5.8	
		No ART, 1st T	1,270	4.8	
		EFV, 1st T	32	15.6	
		No EFV, 1st T	2,001	5.0	
**Joao ** [27] ** 2010**	2002–2007	**All children**	995	6.2	No difference between exposure groups
		Any ART, 1st T	242	6.2	
		Any ART, 2nd T	518	6.8	
		Any ART, 3rd T	208	4.3	
**Fernandez ** [16] ** 2009**	2000–2005	**All children**	623	8.3	No difference between exposure groups
		Any ART, 1st T		8.8	
		Any ART, 2nd–3rd T		7.4	
**Townsend ** [17] ** 2009**	1990–2007	**All children**	8,242	2.8	No difference between exposure groups
		Any ART, 1st T	1,708	3.1	
		Any ART, 2nd–3rd T	5,427	2.7	
		No ART	498	2.8	
**Patel ** [18] ** 2005**	1986–2003	**All children**	3,740	1.5	No difference between exposure groups
		Any ART, 1st T	789	1.8	
		Any ART, 2nd–3rd T	1,184	1.4	
		No ART	1,767	1.4	
		**ART, 1st T**			
		Monotherapy or dual nucleoside	243	1.2	No difference between exposure groups
		Combination ART	546	2.0	
		EFV	19	0.0	

BD, birth defect; EFV, efavirenz; T, trimester.

Our objective was to estimate the prevalence of birth defects in children exposed in utero to ARV drugs taken by their mothers during pregnancy in the large national prospective French Perinatal Cohort (EPF) (Agence Nationale de Recherche sur le Sida et les Hepatites [ANRS] CO1/CO11), and to assess the association of specific birth defects with each in utero ARV drug, taking several known risk factors into account.

## Methods

The study was approved by the Hôpital Cochin Institutional Review Board and the French computer database watchdog commission (Comission Nationale de l'Informatique et des Libertés).

### The French Perinatal Cohort (ANRS CO1/CO11)

Since 1986, EPF has prospectively enrolled pregnant HIV-infected women delivering in 90 centers throughout France [1]. In each participating maternity center, around 95% of all HIV-infected pregnant women were included, with informed consent. No specific recommendation for HIV treatment and obstetric care was made for women included in the cohort, but clinicians were encouraged to follow current French national guidelines, which include trimestrial prenatal ultrasound for all women, whatever their HIV status, and pediatric clinical and biological examinations at birth and 1, 3, 6, 12, and 18–24 mo for children exposed to maternal HIV [4],[20]. No additional ultrasound imaging was done systematically because of the study or because of the HIV status of the mother. Standardized questionnaires were filled out by clinicians, after delivery for pregnancy, and at each visit for children. Variables collected are described below. EPF coverage is estimated to be around 70% of the HIV-infected women in metropolitan France.

### Study Population

**Figure 1:**
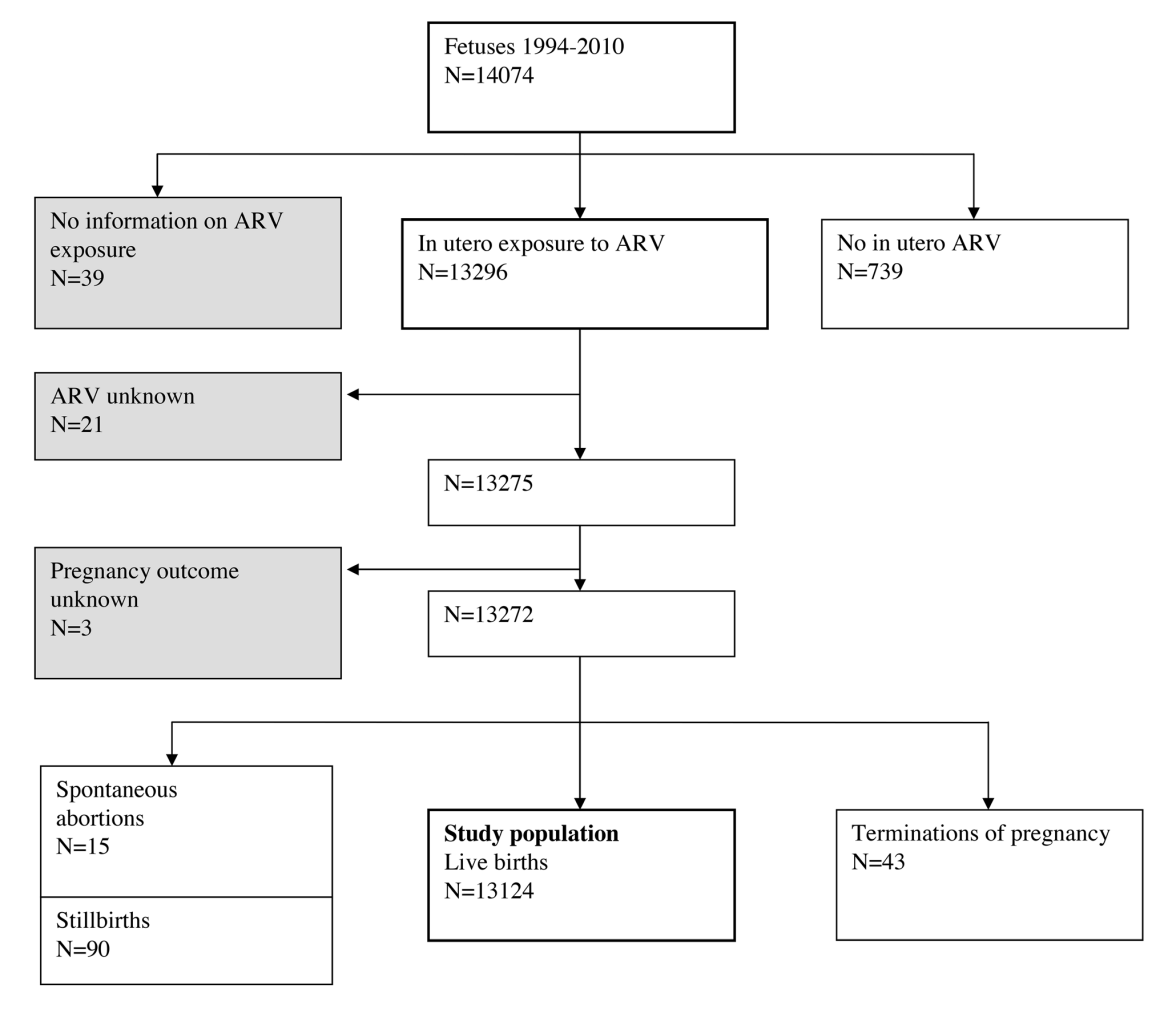
Study population: French Perinatal Cohort (ANRS CO1/CO11).

Among 14,074 fetuses of 13,761 pregnancies included in EPF from 1 January 1994 to 31 December 2010, the main analysis concerned all live births exposed to ART during pregnancy (*n = *13,124) (Figure 1). The small group of women not receiving ART was excluded because it could not be a valid reference group: failing to take ART was related to being socially marginalized, with inadequate access to care and follow-up, as previously reported [21]. We also excluded 43 terminations of pregnancy (TOPs) for fetal abnormalities and 90 stillbirths because, until recently, patients were enrolled after 28 wk of gestation, such that most TOPs and many stillbirths were not represented in EPF. TOPs and stillbirths were added subsequently in sensitivity analyses. No study participants, except 288 women in a collaborative ancillary analysis [14], had been previously included in any published study on birth defects.

### Variables

All clinical events in infants were recorded at each visit (at birth, and at 1, 3, 6, 12, and 18–24 mo). We first coded the birth defects with International Classification of Diseases (ICD-10) codes. We then used EUROCAT inclusion criteria and guidelines in order to assess the overall prevalence of anomalies and to classify them in different organ systems [22],[23]. To facilitate comparison with other studies, we also coded birth defects according to the modified MACDP classification used by the APR [13],[24],[25], which considered as cases children presenting one major defect and/or two conditional defects. In both classifications, each child is counted only once per organ system, even if several defects included in the same organ system are described. The use of both classifications was decided a priori; the EUROCAT classification was used for the primary analysis, and the modified MACDP classification for the secondary analysis.

Maternal variables included age, geographical origin, intravenous drug use (IDU), gravidity, parity, CD4 cell count, and HIV-1 viral load closest to the delivery, i.e., the day of delivery or in the 7 d after delivery. Alcohol and tobacco use were recorded only after 2005, and data on concomitant medications were not available in the cohort.

All ART combinations administered during pregnancy were recorded, with the dates when started and stopped. For each drug (called “index drug”), exposure status was categorized as follows: (1) unexposed to the index drug, but exposed to other drugs (control group), (2) exposed to the index drug since conception or the first trimester, or (3) exposed to the index drug only since the second or third trimester. Another categorization was used in sensitivity analyses concerning only first-trimester exposure: (1) unexposed to any ART in the first trimester, i.e., ART was initiated during second or third trimester (control group), (2) exposed to the index drug in the first trimester, (3) exposed to another drug in the first trimester of pregnancy. Since 1994, the standard of care changed from zidovudine monotherapy (1994–1996), to dual nucleoside reverse transcriptase inhibitors (NRTIs) (1997–1999), to triple-combination drug regimens (selected patients for 2000–2004, and all pregnant women after 2004). We tested 18 ARV drugs for the analysis on overall birth defects and three combinations of drugs: any NRTI, any non-nucleoside reverse transcriptase inhibitor (NNRTI), and any protease inhibitor (PI).

Neonatal variables used in the analysis included gender, gestational age, birth weight, and HIV infection status.

### Statistical Analysis

The prevalence of birth defects was estimated and compared according to various maternal and neonatal characteristics, using the χ^2^ test and Fisher's exact test, as appropriate.

We first studied associations between overall birth defects and each ARV drug using univariate and multivariate logistic regression. Multivariate models included IDU, geographical origin, maternal age, and maternity center. These variables were selected because they were used in the literature and were available for the whole study period. Year of birth was not included in the main analyses because of collinearity with prevention of mother-to-child transmission (PMTCT) strategies; however, it was adjusted for in sensitivity analyses. Alcohol and tobacco use could not be included as adjustment variables because they were not available for the whole study period.

We then studied specific associations between birth defects by organ system and (1) efavirenz, zidovudine, didanosine, and nelfinavir, on the basis of prior studies, and (2) any other ARV drugs associated with overall birth defects at *p*≤0.20 in previous multivariate analysis (these drugs being zalcitabine, lamivudine, emtricitabine, any NNRTI, and indinavir). In order to evaluate the independent effects of drugs often used in combinations, such as zidovudine with lamivudine, we also adjusted for all drugs found to be associated with the type of birth defect under consideration, with a univariate *p*≤0.10, in either the EUROCAT or the MACDP classification.

For each drug considered, the primary analysis compared neonates exposed in the first trimester and those exposed during the second or third trimester with neonates not exposed to the drug during the whole pregnancy (control group). Women with missing data for any adjustment variables were excluded from multivariate analysis, since they did not exceed 2% of the study population for each variable. Correction for multiple comparisons was not performed because the analyses were based on hypotheses emanating from previous findings in the literature and the robustness of the findings of the current study.

Several sensitivity analyses were conducted: (1) exclusion of IDU, (2) inclusion of TOPs and stillbirths, (3) not considering birth defects diagnosed beyond the first week or 6 mo postnatally, (4) using alternative categories for ART exposure status (unexposed to any ART in the first trimester as control group), (5) excluding infants exposed to more than one combination of ART during pregnancy, (6) using more or less parsimonious multivariate models and, particularly, including year of birth, CD4 cell count, or maternal viral load closest to delivery as adjustment variables.

A two-sided *p*<0.05 was taken as indicating statistical significance. Data were analyzed using Stata 11.0 software (Stata Corp) [26]. No adjustment was used for multiple testing.

For each ARV drug, we calculated the power to detect an association between overall birth defects and first-trimester exposure (compared with no exposure during pregnancy). Power to show an odds ratio (OR) of 1.5 was >85% for zidovudine, lamivudine, ritonavir, and lopinavir, and was >70% for didanosine, stavudine, abacavir, tenofovir, and nevirapine (data shown in Table 2). For all these drugs, power was >95% to detect an OR of 2.

**Table 2:** Association between overall birth defects and antiretroviral drugs (French Perinatal Cohort [ANRS CO1/CO11]).

In Utero Exposure	*N* a	Percent with BD	Number with BD^b^	OR^c^	95% CI	*p*-Value^d^	AOR^e^	95% CI	*p*-Value^d^	Power^f^
**Zidovudine**										
Unexposed	2,152	4.0	86	1		0.12	1		0.046	88%
1st T	3,267	5.1	165	1.28	0.98–1.67		1.39	1.06–1.83		
2nd–3rd T	7,493	4.3	322	1.08	0.85–1.38		1.16	0.90–1.51		
Missing	212	0.9	2							
**Didanosine**										
Unexposed	11,651	4.3	500	1		0.01	1		0.02	75%
1st T	927	6.3	58	1.49	1.12–1.97		1.44	1.08–1.92		
2nd–3rd T	529	3.2	17	0.74	0.45–1.21		0.77	0.47–1.27		
Missing	17	0	0							
**Zalcitabine**										
Unexposed	13,010	4.4	567	1		0.2	1		0.07	15%
1st T	103	7.8	8	1.85	0.89–3.82		2	0.94–4.25		
2nd–3rd T	11	0	0	NA			NA			
**Lamivudine**										
Unexposed	3,734	4.0	148	1		0.07	1		0.02	97%
1st T	3,772	5.0	190	1.29	1.03–1.60		1.37	1.06–1.73		
2nd–3rd T	5,398	4.3	234	1.1	0.89–1.35		1.26	1.01–1.57		
Missing	220	1.4	3							
**Stavudine**										
Unexposed	12,127	4.3	520	1		0.17	1		0.43	71%
1st T	819	5.6	46	1.33	0.97–1.81		1.18	0.86–1.63		
2nd–3rd T	169	5.3	9	1.26	0.64–2.47		1.34	0.67–2.67		
Missing	9	0	0							
**Abacavir**										
Unexposed	11,985	4.4	526	1		0.69	1		0.78	76%
1st T	920	4.7	43	1.07	0.78–1.47		1.01	0.73–1.41		
2nd–3rd T	184	3.3	6	0.73	0.32–1.66		0.74	0.32–1.71		
Missing	35	0	0							
**Tenofovir**										
Unexposed	12,043	4.5	536	1		0.51	1		0.3	72%
1st T	823	3.6	30	0.81	0.56–1.18		0.75	0.51–1.10		
2nd–3rd T	208	3.8	8	0.86	0.42–1.75		0.82	0.40–1.69		
Missing	50	2.0	1							
**Emtricitabine**										
Unexposed	12,420	4.5	553	1		0.07	1		0.04	55%
1st T	552	2.5	14	0.56	0.33–0.96		0.52	0.30–0.90		
2nd–3rd T	118	5.9	7	1.35	0.63–2.92		1.38	0.63–3.02		
Missing	34	2.9	1							
**Any NRTI**										
Unexposed	176	2.3	4	1		0.04	1		0.09	14%
1st T	5,288	4.9	261	2.23	0.82–6.06		2.36	0.86–6.47		
2nd–3rd T	7,375	4.2	307	1.87	0.69–5.07		2.04	0.75–5.59		
Missing	285	1.1	3							
**Nevirapine**										
Unexposed	11,936	4.4	521	1		0.94	1		0.82	71%
1st T	819	4.5	37	1.03	0.74–1.46		1	0.71–1.42		
2nd–3rd T	342	4.7	16	1.07	0.64–1.79		1.18	0.70–2.00		
Missing	27	3.7	1							
**Efavirenz**										
Unexposed	12,729	4.4	554	1		0.42	1		0.7	41%
1st T	372	5.4	20	1.25	0.79–1.98		1.16	0.73–1.85		
2nd–3rd T	17	5.9	1	1.37	0.18–10.4		1.83	0.23–14.5		
Missing	6	0	0							
**Any NNRTI**										
Unexposed	11,587	4.3	504	1		0.86	1		0.78	84%
1st T	1161	4.7	54	1.07	0.80–1.43		1.02	0.76–1.37		
2nd–3rd T	343	4.7	16	1.08	0.65–1.79		1.21	0.72–2.03		
Missing	33	3.0	1							
**Amprenavir**										
Unexposed	13,069	4.4	573	1		0.56	1		0.96	6%
1st T	23	0	0	NA			NA			
2nd–3rd T	32	6.3	2	1.45	0.35–6.10		0.96	0.22–4.14		
**Ritonavir**										
Unexposed	7,808	4.6	362	1		0.39	1		0.45	97%
1st T	2,196	4.1	91	0.89	0.70–1.12		0.86	0.67–1.10		
2nd–3rd T	2,891	4.1	119	0.88	0.71–1.09		0.92	0.74–1.15		
Missing	229	1.3	3							
**Saquinavir**										
Unexposed	12,403	4.4	542	1		0.21	1		0.31	35%
1st T	308	6.2	19	1.44	0.89–2.31		1.4	0.86–2.27		
2nd–3rd T	400	3.5	14	0.79	0.46–1.36		0.84	0.48–1.46		
Missing	13	0	0							
**Nelfinavir**										
Unexposed	11,070	4.4	482	1		0.87	1		0.82	60%
1st T	625	4.3	27	0.99	0.67–1.47		0.89	0.59–1.33		
2nd–3rd T	1,419	4.7	66	1.07	0.82–1.39		1.03	0.78–1.36		
Missing	10	0	0							
**Indinavir**										
Unexposed	12,492	4.3	540	1		0.01			0.03	38%
1st T	350	7.7	27	1.85	1.24–2.77		1.66	1.09–2.53		
2nd–3rd T	275	2.9	8	0.66	0.33–1.35		0.69	0.33–1.44		
Missing	7	0	0							
**Atazanavir**										
Unexposed	12,591	4.4	560	1		0.19			0.18	47%
1st T	447	2.7	12	0.59	0.33–1.06		0.58	0.32–1.05		
2nd–3rd T	66	4.5	3	1.02	0.32–3.27		1.23	0.38–4.01		
Missing	20	0	0							
**Lopinavir**										
Unexposed	9,225	4.4	406	1		0.67	1		0.42	88%
1st T	1,333	4.1	55	0.93	0.70–1.25		0.92	0.68–1.23		
2nd–3rd T	2,371	4.7	112	1.08	0.87–1.33		1.13	0.90–1.41		
Missing	195	1.0	2							
**Fosamprenavir**										
Unexposed	12,873	4.4	564	1		0.73			0.77	22%
1st T	172	5.2	9	1.21	0.61–2.37		1.13	0.57–2.25		
2nd–3rd T	72	2.8	2	0.62	0.15–2.55		0.63	0.15–2.61		
Missing	7	0.0	0							
**Any PI**										
Unexposed	5,642	4.6	257	1		0.81			0.69	99%
1st T	3,125	4.4	139	0.98	0.79–1.20		0.91	0.73–1.13		
2nd–3rd T	4,110	4.3	176	0.94	0.77–1.14		0.94	0.77–1.16		
Missing	247	1.2	3							

Missing data were excluded from all statistical tests.

a Total number of patients exposed in each category.

b Number of birth defects observed among *N* patients of the category.

c ORs obtained by univariate logistic regression.

d Global *p*-value for exposure to each drug, including the three categories (no exposure, exposure in the first trimester, and exposure in the 2nd or 3rd trimester).

e AOR obtained by multivariate logistic regression, adjusted systematically for mother's age, geographical origin, IDU, and maternity center.

f Power for an OR of 1.5 concerning exposure in the first trimester to each drug as compared to no exposure to this drug.

BD, birth defect; NA, not applicable/no child in this category; T, trimester.

## Results

The analysis was conducted in 13,124 live-born children, born between 1 January 1994 and 31 December 2010, among whom 42% (*n = *5,388) were exposed to ART in the first trimester of pregnancy. PMTCT strategies varied over time, and the proportion of infants exposed to ART in the first trimester increased from 19% in 1994–1996 to 52% in 2005–2010. The evolution in time of types of treatment and trimesters of exposure are presented in Table 3. The median maternal age was 31 y, most women were from sub-Saharan Africa (61%) and parous (62%), and very few were intravenous drug users (2%). Most women had a good immunovirological status (CD4≥350 cells/ml and viral load <400 copies/ml; Table 4). Data at birth was collected for the newborns of all women enrolled. Median follow-up of children was 19 mo (interquartile range 12–24 mo).

**Table 3:** Evolution of antiretroviral drugs during the study period.

ART	Time Period				*p*-Value
	1994–1996	1997–1999	2000–2004	2005–2010	
**Any ART (** ***n*** **)**	1,055	1,884	4,555	5,342	
1st T (percent)	19.1	35.7	38.5	51.7	<0.001
2nd–3rd T (percent)	80.9	64.3	61.5	48.3	
**Monotherapy ZDV (** ***n*** **)**	1,002	376	817	63	
1st T (percent)	17.0	8.0	3.3	7.9	<0.001
2nd–3rd T (percent)	83.0	92.0	96.7	92.1	
**Dual-therapy NRTI (** ***n*** **)**	18	581	721	120	
1st T (percent)	50.0	38.0	24.8	13.3	<0.001
2nd–3rd T (percent)	50.0	62.0	75.2	86.7	
**Combination ART (** ***n*** **)**	3	307	2,745	5,084	
1st T (percent)	100.0	79.8	51.9	53.0	<0.001
2nd–3rd T (percent)	0	20.2	48.1	47.0	
**ZDV (** ***n*** **)**	1,055	1,885	4,559	5,413	
No ZDV (percent)	0	8.7	13.6	25.3	<0.001
1st T (percent)	18.4	22.9	23.4	29.1	
2nd–3rd T (percent)	81.6	68.4	63.0	45.6	
**EFV (** ***n*** **)**	1,080	1,889	4,567	5,582	
No EFV (percent)	100	99.6	96.3	96.2	<0.001
1st T (percent)	0	0.3	3.4	3.7	
2nd–3rd T (percent)	0	0.1	0.2	0.1	

EFV, efavirenz; T, trimester; ZDV, zidovudine.

**Table 4:** Maternal and neonatal characteristics, and associations with overall birth defects, according to the EUROCAT classification (French Perinatal Cohort [ANRS CO1/CO11]).

Characteristic	*N* a	Percent with BD	Number with BD^b^	OR	95% CI	*p*-Value^c^
**Maternal age (years)**						
<25	1,502	3.4	51	1		0.046
25–34	7,800	4.3	337	1.28	0.95–1.73	
>35	3,781	4.9	186	1.47	1.07–2.02	
Missing	41	2.4	1			
**Geographical origin**						
France	2,818	4.7	132	1		0.31
Sub-Saharan Africa	7,920	4.2	331	0.89	0.72–1.09	
Other	2,225	4.8	107	1.03	0.79–1.33	
Missing	161	3.1	5			
**IDU during pregnancy**						
No	12,622	4.4	557	1		0.69
Yes	264	4.9	13	1.12	0.64–1.97	
Missing	238	2.1	5			
**CD4 count (cells/mm^3^)**						
>350	7,605	4.6	353	1		0.56
200–350	2,361	4.4	103	0.93	0.75–1.17	
<200	1,059	5.2	55	1.12	0.84–1.51	
Missing	2,099	3.0	64			
**Viral load (copies/ml)**						
<400	8,710	4.5	391	1		0.76
400–1,000	668	5.4	36	1.21	0.85–1.72	
1,000–10,000	1,292	4.6	60	1.04	0.78–1.36	
>10,000	649	4.6	30	1.03	0.71–1.51	
Missing	1,805	3.2	58			
**Parity**						
Nulliparous	4,909	4.3	209	1		0.52
Parous	8,137	4.5	366	1.06	0.89–1.26	
Missing	78	0.0	0			
**Pregnancy**						
Singleton	12,545	4.4	547	1		0.59
Multiple	579	4.8	28	1.12	0.76–1.65	
**Gender of neonate**						
Male	6,567	4.9	322	1		0.01
Female	6,265	4.0	249	0.8	0.68–0.95	
Missing	292	1.4	4			
**Neonate HIV-infected**						
Yes	174	2.9	5	0.62	0.25–1.52	0.29
No	11,777	4.5	535	1		
Undetermined	1,173	3.0	35			
**Year of birth**						
1994–1996	1,080	3.1	34	0.8	0.56–1.16	0.001
1997–1999	1,889	5.7	107	1.49	1.17–1.88	
2000–2004	4,567	4.8	217	1.23	1.02–1.50	
2005–2010	5,588	3.9	217	1		
**Mode of delivery** d						
Vaginal	5,076	3.9	197	1		0.002
Cesarean	2,388	5.1	352	1.33	1.11–1.59	
Missing	1,144	2.3	26			
**Premature delivery (<37 wk)** a						
Yes	1,901	6.6	126	1.69	1.38–2.07	<0.001
No	11,154	4.0	449	1		
Missing	69	0.0	0			
**Low birth weight (<2,500 g)** d						
Yes	2,127	6.9	146	1.78	1.47–2.16	<0.001
No	10,713	4.0	426	1		
Missing	284	1.1	3			

a Total patients in each category.

b Number of birth defects among the *N* patients of the category.

c χ^2^ test or Fisher's exact test; missing data excluded.

d Association with mode of delivery, premature delivery and low birth weight could be a consequence but not a risk factor for birth defects, and thus were not included in the multivariate analysis.

### Overall Birth Defects

The overall birth defect rate was 4.4% (95% CI 4.0%–4.7%) (*n = *575/13,124), according to the EUROCAT classification and 7.0% (95% CI 6.5%–7.4%) (*n = *914/13,124) according to the MACDP classification. The rate increased from 1994–1996 to 1997–1999 and then decreased slightly afterwards. The presence of a birth defect was significantly associated with male gender and higher maternal age. Neonates with birth defects were more frequently born by cesarean section, preterm, and with low birth weight (Table 4).

**Figure 2:**
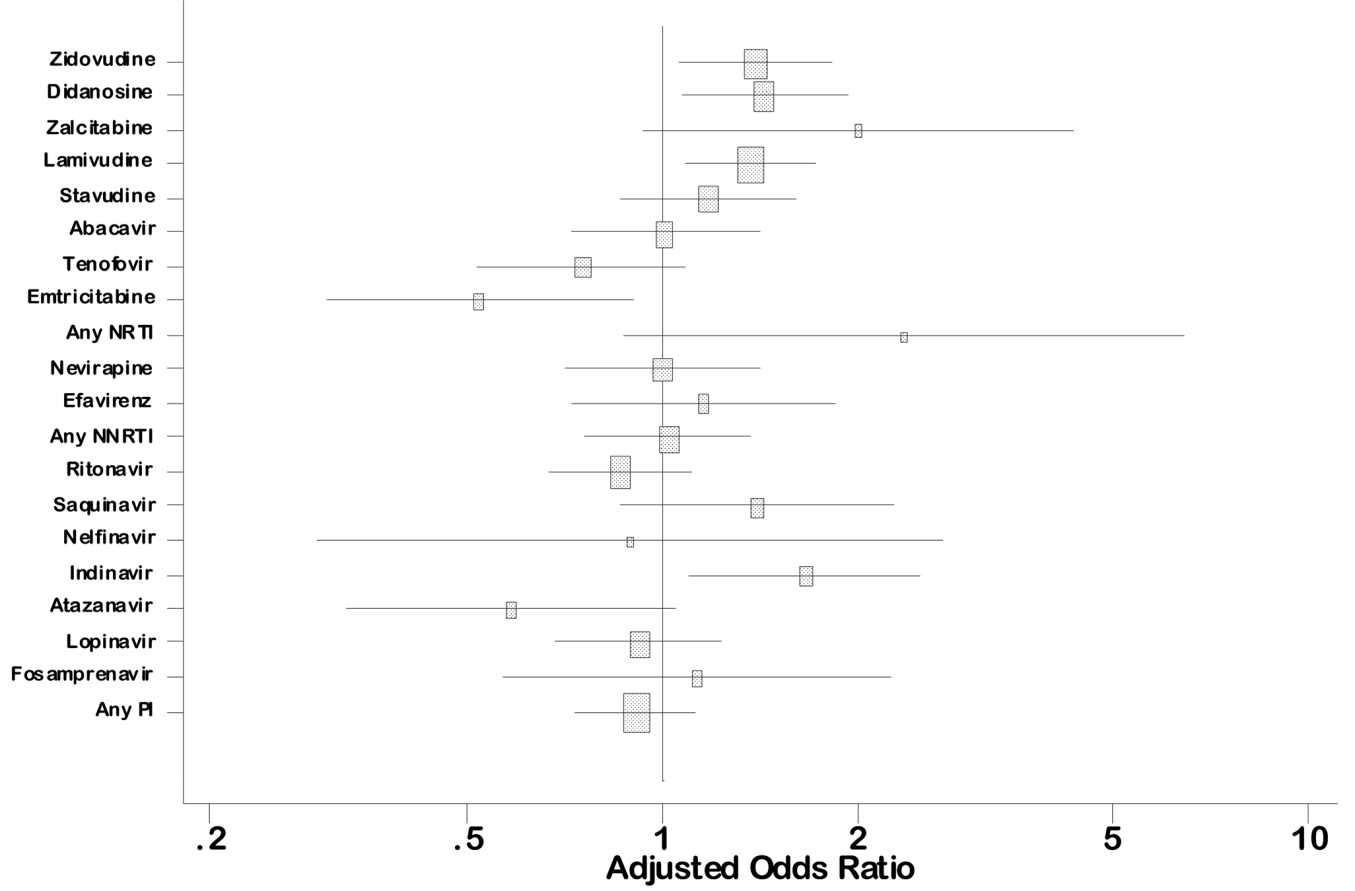
Association between overall birth defects and first trimester antiretroviral drug exposure (French Perinatal Cohort [ANRS CO1/CO11]): multivariate analysis. Squares indicate AORs for exposure in the first trimester versus no exposure to the drug, adjusted on IDU, geographical origin, maternal age, and maternity center. Lines indicate 95% confidence intervals and square areas are proportional to the power for an OR of 1.5. Total number of birth defects = 575/13,124. Numbers for each ARV drug are shown in Table 3.

Associations with ARV drugs are presented using the EUROCAT classification, and studied in sensitivity analyses for the MACDP classification. Using the EUROCAT classification, overall birth defects were significantly associated with zidovudine in the first trimester, compared with no zidovudine during pregnancy (5.1% for *n = *3,267 children exposed in the first trimester versus 4.0% for *n = *2,152 children not exposed to zidovudine, AOR = 1.39 [95% CI 1.06–1.83], *p<*0.05), as well as with didanosine (6.3%, *n = *927, for first-trimester exposure versus 4.3%, *n = *11,651, for unexposed, AOR = 1.44 [95% CI 1.08–1.92], *p = *0.02), lamivudine (5.0%, *n = *3,772, for first-trimester exposure versus 4.0%, *n = *3,734, for unexposed, AOR = 1.37 [95% CI 1.06–1.76], *p = *0.02) and indinavir (7.7%, *n = *350, for first-trimester exposure versus 4.3, *n = *12,492, for unexposed, AOR = 1.66 [95% CI 1.09–2.53], *p = *0.03) (Figure 2; Table 2). Defects according to the MACDP classification were associated with the same four drugs, as well as with zalcitabine (AOR = 2.16 [95% CI 1.17–4.00], *p = *0.01) and any kind of NNRTI (AOR = 1.33 [95% CI 1.07–1.66], *p = *0.03) (Table 5). These associations were independent of IDU, geographical origin, maternal age, and maternity center.

**Table 5:** Association between overall birth defects and antiretroviral drugs according to MACDP classification (French Perinatal Cohort [ANRS CO1/CO11]).

In Utero Exposure	*N* a	Percent with BD	Number with BD^b^	OR^c^	95% CI	*p*-Value^d^	AOR^e^	95% CI	*p*-Value^d^
**Zidovudine**									
Unexposed	2,152	6.4	137	1		0.007	1		0.002
1st T	3,267	8.3	271	1.32	1.07–1.63		1.41	1.13–1.76	
2nd–3rd T	7,493	6.7	502	1.05	0.87–1.28		1.1	0.89–1.35	
**Didanosine**									
Unexposed	11,651	6.8	796	1		0.002	1		0.006
1st T	927	9.7	90	1.46	1.16–1.84		1.4	1.11–1.78	
2nd–3rd T	529	5.3	28	0.76	0.51–1.12		0.77	0.52–1.14	
**Zalcitabine**									
Unexposed	13,010	6.9	900	1		0.02	1		0.01
1st T	103	13.6	14	2.11	1.20–3.72		2.16	1.17–4.00	
2nd–3rd T	11	0	0	NA			NA		
**Lamivudine**									
Unexposed	3,734	6.3	237	1		0.006	1		<0.001
1st T	3,772	8.2	308	1.31	1.10–1.56		1.43	1.18–1.72	
2nd–3rd T	5,398	6.7	362	1.06	0.90–1.26		1.2	1.01–1.44	
**Stavudine**									
Unexposed	12,127	6.9	838	1		0.48	1		0.75
1st T	819	7.9	65	1.16	0.89–1.50		1.07	0.82–1.41	
2nd–3rd T	169	5.9	10	0.84	0.44–1.60		0.84	0.43–1.61	
**Abacavir**									
Unexposed	11,985	7	833	1		0.48	1		0.51
1st T	920	7.7	71	1.12	0.87–1.44		1.09	0.84–1.42	
2nd–3rd T	184	5.4	10	0.77	0.40–1.46		0.73	0.38–1.41	
**Tenofovir**									
Unexposed	12,043	7.1	854	1		0.05	1		0.03
1st T	823	6.2	51	0.89	0.66–1.18		0.8	0.59–1.08	
2nd–3rd T	208	3.4	7	0.46	0.21–0.97		0.42	0.19–0.90	
**Emtricitabine**									
Unexposed	12,420	7.1	877	1		0.22	1		0.13
1st T	552	5.6	31	0.81	0.56–1.16		0.74	0.51–1.07	
2nd–3rd T	118	4.2	5	0.58	0.24–1.43		0.55	0.22–1.36	
**Any NRTI**									
Unexposed	176	4	7	1		<0.001	1		0.002
1st T	5,288	8	425	2.12	0.99–4.53		2.13	0.98–4.61	
2nd–3rd T	7,375	6.5	476	1.68	0.78–3.58		1.69	0.78–3.65	
**Nevirapine**									
Unexposed	11,936	6.9	821	1		0.1	1		0.11
1st T	819	8.7	71	1.28	0.99–1.65		1.31	1.00–1.69	
2nd–3rd T	342	5.6	19	0.79	0.50–1.27		0.88	0.54–1.41	
**Efavirenz**									
Unexposed	12,729	6.9	878	1		0.21	1		0.31
1st T	372	9.4	35	1.4	0.98–1.99		1.32	0.92–1.91	
2nd–3rd T	17	5.9	1	0.84	0.11–6.34		1.06	0.13–8.31	
**Any NNRTI**									
Unexposed	11,587	6.8	789	1		0.02	1		0.03
1st T	1,161	8.9	103	1.33	1.07–1.64		1.33	1.07–1.66	
2nd–3rd T	343	5.5	19	0.8	0.50–1.28		0.89	0.55–1.44	
**Amprenavir**									
Unexposed	13,069	7	909	1		0.45	1		0.3
1st T	23	0	0	NA			NA		
2nd–3rd T	32	15.6	5	2.47	0.95–6.43		1.68	0.63–4.50	
**Ritonavir**									
Unexposed	7,808	7.4	576	1		0.25	1		0.16
1st T	2,196	7	153	0.95	0.79–1.14		0.91	0.75–1.11	
2nd–3rd T	2,891	6.2	179	0.83	0.70–0.99		0.84	0.70–1.01	
**Saquinavir**									
Unexposed	12,403	7	863	1		0.46	1		0.58
1st T	308	8.4	26	1.23	0.82–1.84		1.21	0.80–1.84	
2nd–3rd T	400	6	24	0.85	0.56–1.29		0.9	0.59–1.39	
**Nelfinavir**									
Unexposed	11,070	6.8	757	1		0.29	1		0.58
1st T	625	8.5	53	1.26	0.94–1.68		1.17	0.87–1.58	
2nd–3rd T	1419	7.3	103	1.06	0.86–1.31		1.02	0.82–1.29	
**Indinavir**									
Unexposed	12,492	6.9	859	1		0.006	1		0.04
1st T	350	11.4	40	1.74	1.24–2.44		1.52	1.07–2.17	
2nd–3rd T	275	5.5	15	0.78	0.46–1.32		0.76	0.44–1.31	
**Atazanavir**									
Unexposed	12,591	7	883	1		0.58	1		0.57
1st T	447	6.3	28	0.88	0.60–1.30		0.85	0.57–1.26	
2nd–3rd T	66	4.5	3	0.63	0.20–2.01		0.65	0.20–2.11	
**Lopinavir**									
Unexposed	9,225	7.3	669	1		0.36	1		0.4
1st T	1,333	6.4	85	0.88	0.70–1.11		0.86	0.67–1.09	
2nd–3rd T	2,371	6.6	156	0.91	0.76–1.08		0.93	0.77–1.12	
**Fosamprenavir**									
Unexposed	12,873	7	896	1		0.41	1		0.61
1st T	172	8.7	15	1.27	0.75–2.17		1.18	0.68–2.03	
2nd–3rd T	72	4.2	3	0.58	0.18–1.84		0.62	0.19–2.00	
**Any PI**									
Unexposed	5,642	7.1	403	1		0.19	1		0.45
1st T	3,125	7.6	237	1.07	0.90–1.26		1.01	0.85–1.21	
2nd–3rd T	4,110	6.5	267	0.9	0.77–1.06		0.91	0.77–1.08	

Missing data were excluded from all statistical tests.

a Total of patients exposed in each category.

b Number of birth defects observed among N patients of the category.

c OR obtained by univariate logistic regression.

d For exposure to each drug, including the three categories (no exposure, exposure in the first trimester and in the second or third trimester).

e AOR obtained by multivariate logistic regression.

BD, birth defect; NA, not applicable/no child in this category; T, trimester.

### Birth Defects by Organ System

Exposure to efavirenz during the first trimester was not found to be associated with birth defects overall in the EUROCAT classification (5.4%, *n = *20/372, AOR = 1.16 [95% CI 0.73–1.85], *p = *0.70) (Figure 2; Table 2). However, there was a statistically significant association between neurological birth defects and efavirenz in the first trimester in the secondary analysis using the modified MACDP classification (1.1% among 372 children exposed to efavirenz in the first trimester versus 0.4% among 12,729 children unexposed, AOR = 3.0 [95% CI 1.1–8.5], *p = *0.04, absolute risk difference +0.7% [95% CI +0.07%; +1.3%]) (Table 6). This association did not reach significance in the primary analysis using the EUROCAT classification (AOR = 2.1 [95% CI 0.7–5.9], *p = *0.16). The four neurological defects, according to MACDP, reported in children exposed to efavirenz in the first trimester were ventricular dilatation with anomalies of the white substance, partial agenesis of the corpus callosum, subependymal cyst, and pachygyria. Congenital infection or associated non-neurological defects were not reported for these four children. Efavirenz was not associated with other birth defects.

**Table 6:** Association between birth defects by organ system and ARV drug exposures (French Perinatal Cohort [ANRS CO1/CO11]).

Type of BD	In Utero Exposure	Percent with BD	*n*/*N* a	EUROCAT									MACDP					
				LBs Only						LBs, TOPs, and SBs			LBs Only			LBs, TOPs, and SBs		
				OR	95% CI	*p*-Value	AOR	95% CI	*p*-Value	AOR	95% CI	*p*-Value	AOR	95% CI	*p*-Value	AOR	95% CI	*p*-Value
**Central nervous system**	**EFV**					0.13			0.16			0.22			0.04			
	Unexposed	0.4	56/12,729	1			1			1			1			1		0.07
	1st T	1.1	4/372	2.5	0.9–6.8		2.1	0.7–5.9		1.9	0.7–5.4		3.0	1.1–8.5		2.7	0.9–7.5	
	2nd–3rd T	0.0	0/17	NA			NA			NA			NA			NA		
	**EFV, 1st T**					0.31			0.30			0.41						
	No drugs, 1st T	0.4	32/7,448	1			1			1			1		0.08	1		0.14
	EFV, 1st T	1.1	4/372	2.5	0.9–7.2		1.8	0.6–5.4		1.7	0.6–5.1		2.5	0.8–7.5		2.3	0.8–6.8	
	Other, 1st T	0.5	23/5,012	1.1	0.6–1.8		0.8	0.4–1.4		0.8	0.5–1.5		0.7	0.4–1.4		0.8	0.4–1.4	
**Heart and circulatory system**	**ZDV**					<0.001			0.003			0.005			0.002			0.004
	Unexposed	1.1	23/2,152	1			1			1			1			1		
	1st T	2.3	74/3,267	2.1	1.3–3.4		2.2	1.3–3.7		2.1	1.3–3.5		2.4	1.4–4.1		2.3	1.4–3.9	
	2nd–3rd T	1.1	84/7,493	1.1	0.7–1.7		1.1	0.6–1.9		1.1	0.6–1.9		1.4	0.8–2.4		1.4	0.8–2.4	
	**ZDV, 1st T**					<0.001			0.003			0.006			0.003			0.006
	No drugs, 1st T	1.1	83/7,448	1			1			1			1			1		
	ZDV, 1st T	2.3	74/3,267	2.1	1.5–2.8		2.1	1.3–3.6		2.0	1.2–3.4		1.9	1.2–3.2		1.8	1.1–3.0	
	Other, 1st T	1.1	24/2,117	1.0	0.6–1.6		1.1	0.6–1.9		1.1	0.6–1.9		0.9	0.5–1.6		0.9	0.5–1.6	
**Head and neck defects**	**DDI**					0.06			0.07			0.07			0.049			0.05
	Unexposed	0.2	18/11,651	1			1			1			1			1		
	1st T	0.5	5/927	3.5	1.3–9.5		3.4	1.1–10.4		3.3	1.1–10.3		1.9	1.1–3.3		1.9	1.1–3.3	
	2nd–3rd T	0.4	2/529	2.5	0.6–10		2.7	0.6–12.0		2.7	0.6–12.1		0.6	0.2–1.9		0.6	0.2–1.9	
	**DDI, 1st T**					0.12			0.12			0.13			0.20			0.20
	No drugs, 1st T	0.2	12/7448	1			1			1			1			1		
	DDI, 1st T	0.5	5/927	3.4	1.2–9.6		2.9	0.8–10.9		2.8	0.7–10.7		1.5	0.8–2.9		1.5	0.8–2.9	
	Other, 1st T	0.2	8/4,457	1.1	0.5–2.7		NA			NA			NA			NA		
	**IND**					0.01			0.04			0.04			0.47			0.49
	Unexposed	0.2	22/12,470	1			1			1			1			1		
	1st T	0.9	3/350	4.9	1.5–16		3.8	1.1–13.8		3.8	1.0–13.7		1.6	0.7–3.5		1.6	0.7–3.5	
	2nd–3rd T	0	0/275	NA			NA			NA			0.8	0.2–3.1		0.8	0.2–3.1	
	**IND, 1st T**					0.02			0.04			0.04			0.38			0.34
	No drugs, 1st T	0.2	12/7,448	1			1			1			1				1	
	IND, 1st T	0.9	3/350	5.4	1.5–19		4.0	1.1–15.0		4.0	1.1–14.9		1.5	0.6–3.2		1.5	0.7–3.3	
	Other, 1st T	0.2	10/5,038	1.2	0.5–2.9		NA			NA			NA				NA	

All organ system defects significantly associated with ART in multivariate main analysis with either EUROCAT or MACDP classification are presented in this table. AORs obtained by multivariate logistic regression adjusted systematically for maternal age, geographical origin, IDU, and type of maternity center (ranged from 1 to 3 according to the presence of a pediatrician on site, a neonatology unit, or a neonatal intensive care unit), and for all other drugs associated in univariate analysis with *p<*0.10 to the organ system considered in either classification (central nervous system defects adjusted for didanosine; heart defects adjusted for indinavir, emtricitabine, lamivudine, and zalcitabine; head and neck defects adjusted for zidovudine, zalcitabine, lamivudine, emtricitabine and efavirenz). Univariate analysis showing the association between birth defects by organ system and all drugs evaluated is presented in Table 3. *p*-Values are global *p*-value for the three categories in each type of exposure.

a Number of birth defects/total in exposure category.

BD, birth defect; DDI, didanosine; EFV, efavirenz; IND, indinavir; LB, live birth; NA, not applicable/no child in this category; SB, stillbirth; T, trimester; ZDV, zidovudine.

Exposure to zidovudine during the first trimester was associated with CHDs according to the EUROCAT classification (2.3% among the 3,267 children exposed to zidovudine in the first trimester versus 1.1% among the 2,152 children unexposed to zidovudine, AOR = 2.2 [95% CI 1.3–3.7], *p = *0.003) (Table 6). The absolute risk difference attributed to zidovudine was therefore +1.2% (95% CI +0.5; +1.9%). CHDs according to EUROCAT classification were also associated with zalcitabine, lamivudine, and indinavir in the univariate analysis, but after adjustment for other concomitant ARV drugs, the association did not reach significance. Six children exposed to emtricitabine during the second or third trimester were diagnosed with a CHD (5.1%, *n = *6/118, versus 1.4% among the 12,420 children unexposed to emtricitabine, AOR = 4.5 [95% CI 1.9–10.9], *p = *0.001), but four of them were also exposed to zidovudine during the first trimester. The association between zidovudine and heart defects remained the same when (1) limited to diagnoses made in the first 6 mo or in the first week of life, or to infants exposed to only one combination of ART during pregnancy, (2) adjusted for prematurity, parity, CD4 count, year of birth, and gender (AOR for zidovudine = 2.2 [95% CI 1.3–3.6], *p = *0.003), (3) using alternate categorization for zidovudine exposure (AOR = 2.1 [95% CI 1.3–3.6], *p = *0.003, for zidovudine in the first trimester versus no treatment at all during the first trimester), (4) using the MACDP classification (Table 6), and (5) including TOPs and stillbirths. Zidovudine was not found to be associated with other types of birth defects. In the largest multivariate model cited above, the other variables significantly associated with CHDs were maternal age >35 y, prematurity (AOR = 2.5 [95% CI 1.7–3.6]), female gender (AOR = 1.4 [95% CI 1.0–2.0]), and CD4 count <200 cells/ml (AOR = 1.7 [95% CI 1.1–2.7] versus CD4>350 cells/ml). Time period was not independently associated with heart defects (*p = *0.23). Among the 74 children with a diagnosis of CHD and exposed to zidovudine during the first trimester, the most frequent heart defects were ventricular septal defects (*n = *43), atrial septal defects (*n = *13), and persistence of the ductus arteriosus (*n = *9). We found no significant association between heart defects and any of the other ARV drugs included in the multivariate model, which were lamivudine, zalcitabine, emtricitabine, and indinavir. No interaction was found between zidovudine and lamivudine.

Head and neck defects, as described in the EUROCAT guidelines [22],[23], were associated with didanosine (0.5%, *n = *5/927, for first-trimester exposure versus 0.2%, *n = *18/11,651, in the unexposed group, AOR = 3.4 [95% CI 1.1–10.4], *p = *0.04) and with indinavir (0.9%, *n = *3/350, in the exposed versus 0.2%, *n = *22/12,470, in the unexposed group, AOR = 3.8 [95% CI 1.1–13.8], *p = *0.04) (Table 6). Didanosine and indinavir were prescribed to 6.9% and 3% of the women in the study in the last 6 y, respectively.

The association between birth defect by organ system and each ARV drug is described in Table 7. All birth defects among live births are listed Table 8, and birth defects among TOP are listed in Table 9. We found no association between exposure to other ARV drugs and overall birth defects or specific birth defects, including for tenofovir, with 823 children exposed in the first trimester: AOR = 0.75 (95% CI 0.51–1.10), with a power of 72% for an OR of 1.5 (Table 2).

**Table 7:** Univariate analysis: association between birth defects by organ system and antiretroviral drugs.

In Utero Exposure	Central Nervous System						Heart and Circulatory System						Renal and Urinary System						Limb and Musculoskeletal System						Head and Neck (Including Eyes and Ears)					
	EUROCAT (*n = *60)			MACDP (*n = *50)			EUROCAT (*n = *182)			MACDP (*n = *198)			EUROCAT (*n = *53)			MACDP (*n = *57)			EUROCAT (*n = *133)			MACDP (*n = *373)			EUROCAT (*n = *25)			MACDP (*n = *129)		
	OR	95% CI	*p*-Value	OR	95% CI	*p*-Value	OR	95% CI	*p*-Value	OR	95% CI	*p*-Value	OR	95% CI	*p*-Value	OR	95% CI	*p*-Value	OR	95% CI	*p*-Value	OR	95% CI	*p*-Value	OR	95% CI	*p*-Value	OR	95% CI	*p*-Value
**Zidovudine**																														
Unexposed	1		0.96	1		0.47	1		<0.001	1		<0.001	1		0.64	1		0.52	1		0.45	1		0.13	1		0.91	1		0.07
1st T	1.1	0.48–2.51		0.66	0.26–1.66		2.14	1.34–3.44		2.45	1.51–3.98		1.13	0.44–2.87		1.22	0.49–3.07		1.32	0.77–2.27		1.29	0.93–1.79		0.79	0.24–2.59		1.39	0.82–2.33	
2nd–3rd T	1.11	0.54–2.33		1.02	0.49–2.14		1.05	0.66–1.67		1.34	0.84–2.16		1.4	0.62–3.16		1.52	0.68–3.42		1.05	0.64–1.72		1.03	0.76–1.39		0.8	0.29–2.23		0.87	0.53–1.43	
**Didanosine**																														
Unexposed	1		0.09	1		0.77	1		0.82	1		0.81	1		0.57	1		0.87	1		0.06	1		0.07	1		0.06	1		0.03
1st T	2.37	1.16–4.85		1.17	0.42–3.27		1.18	0.69–2.01		1.15	0.69–1.93		1.34	0.53–3.37		1.26	0.50–3.16		1.64	0.95–2.81		1.45	1.02–2.05		3.5	1.30–9.46		1.97	1.18–3.31	
2nd–3rd T	1.38	0.43–4.44		1.54	0.48–4.98		0.96	0.45–2.06		0.88	0.41–1.88		0.47	0.06–3.40		0.88	0.21–3.63		0.38	0.09–1.53		0.74	0.40–1.36		2.45	0.57–10.6		0.6	0.19–1.91	
**Zalcitabine**																														
Unexposed	NA			NA			1		0.04	1		0.009	1		0.44	1		0.47	1		0.96	1		0.97	NA			1		0.06
1st T	NA			NA			2.91	1.06–8.00		3.39	1.36–8.42		2.44	0.33–17.9		2.27	0.31–16.5		0.96	0.13–6.90		1.02	0.32–3.25		NA			3.07	0.96–9.80	
2nd–3rd T	NA			NA			NA			NA			NA			NA			NA			NA			NA			NA		
**Lamivudine**																														
Unexposed	1		0.85	1		0.84	1		0.02	1		0.1	1		0.31	1		0.3	1		0.27	1		0.02	1		0.27	1		0.004
1st T	0.89	0.46–1.71		0.8	0.39–1.67		1.48	1.02–2.25		1.36	0.95–1.95		1.35	0.62–2.95		1.32	0.62–2.80		1.3	0.84–2.01		1.29	0.99–1.68		2.48	0.78–7.91		2.07	1.29–3.31	
2nd–3rd T	0.84	0.46–1.54		0.91	0.47–1.74		0.94	0.64–1.37		0.97	0.68–1.39		1.7	0.84–3.43		1.68	0.85–3.29		0.94	0.61–1.45		0.92	0.71–1.19		1.9	0.61–5.98		1.28	0.79–2.07	
**Tenofovir**																														
Unexposed	1		0.93	1		0.65	1		0.21	1		0.69	1		0.82	1		0.85	1		0.86	1		0.03	1		0.75	1		0.21
1st T	1.04	0.38–2.89		1.27	0.48–3.55		0.79	0.40–1.54		0.8	0.42–1.51		0.88	0.27–2.82		1.1	0.40–3.06		0.94	0.46–1.92		0.87	0.56–1.36		1.27	0.30–5.41		0.59	0.24–1.44	
2nd–3rd T	NA			NA			2.11	0.92–4.83		1.27	0.47–3.46		NA			NA			NA			0.16	0.02–1.15		NA			NA		
**Emtricitabine**																														
Unexposed	1		0.78	1		0.56	1		0.02	1		0.16	1		0.86	1		0.72	1		0.21	1		0.62	NA			1		0.08
1st T	1.18	0.37–3.80		1.44	0.44–4.64		0.65	0.27–1.60		0.59	0.24–1.44		0.88	0.21–3.63		1.25	0.39–4.01		0.52	0.16–1.63		0.87	0.51–1.50		NA			0.35	0.09–1.43	
2nd–3rd T	NA			NA			3.84	1.67–8.85		2.27	0.83–6.22		NA			NA			NA			NA			NA			NA		
**Nevirapine**																														
Unexposed	1		0.12	1		0.37	1		0.16	1		0.46	1		0.12	1		0.09	1		0.32	1		0.03	NA			1		0.19
1st T	1.75	0.75–4.10		1.36	0.49–3.79		0.8	0.41–1.57		0.9	0.49–1.66		0.28	0.04–2.02		0.26	0.04–1.88		1.49	0.82–2.71		1.57	1.10–2.23		NA			1.39	0.74–2.58	
2nd–3rd T	2.81	1.01–7.83		2.45	0.76–7.93		1.94	0.98–3.83		1.58	0.77–3.24		NA			NA			0.15	0.15–2.39		0.63	0.28–1.42		NA			0.3	0.04–2.15	
**Efavirenz**																														
Unexposed	1		0.13	1		0.04	1		0.46	1		0.51	1		0.66	1		0.77	1		0.67	1		0.11	1		0.2	1		0.04
1st T	2.46	0.89–6.82		3	1.07–8.37		1.18	0.52–2.67		0.89	0.36–2.17		0.66	0.09–4.77		1.25	0.30–5.13		0.79	0.25–2.49		1.56	0.93–2.60		2.99	0.70–12.7		2.29	1.11–4.72	
2nd–3rd T	NA			NA			4.48	0.59–34.0		4.08	0.54–30.9		NA			NA			NA			NA			NA			NA		
**Any NNRTI**																														
Unexposed	1		0.03	1		0.1	1		0.19	1		0.47	1		0.13	1		0.28	1		0.44	1		0.01	1		0.84	1		0.05
1st T	2.18	1.10–4.33		2.05	0.96–4.41		0.87	0.51–1.52		0.92	0.55–1.54		0.39	0.09–1.60		0.55	0.17–1.77		1.31	0.76–2.24		1.57	1.15–2.13		0.87	0.20–3.68		1.65	1.00–2.74	
2nd–3rd T	2.96	1.06–8.27		2.61	0.80–8.50		1.94	0.98–3.82		1.56	0.77–3.23		NA			NA			0.59	0.14–2.38		0.63	0.28–1.43		NA			0.31	0.04–2.21	
**Nelfinavir**																														
Unexposed	1		0.29	1		0.16	1		0.73	1		0.68	1		0.4	1		0.36	1		0.84	1		0.51	1		0.87	1		0.89
1st T	1.54	0.55–4.30		0.93	0.22–3.87		1.29	0.69–2.39		1.29	0.71–2.32		1.73	0.62–4.85		1.61	0.58–4.51		0.78	0.32–1.90		1.28	0.82–1.99		0.8	0.11–5.98		1.16	0.54–2.50	
2nd–3rd T	1.7	0.86–3.38		2.06	1.02–4.14		0.97	0.60–1.58		0.94	0.59–1.50		1.53	0.71–3.26		1.6	0.78–3.28		0.96	0.96–1.67		1.1	0.79–1.51		0.71	0.17–3.02		1.09	0.64–1.88	
**Indinavir**																														
Unexposed	1		0.59	1		0.22	1		0.07	1		0.04	1		0.04	1		0.06	1		0.86	1		0.64	1		0.03	1		0.21
1st T	1.92	0.60–6.16		2.29	0.71–7.39		2.13	1.11–4.07		2.16	1.16–4.00		3.92	1.55–9.93		3.61	1.43–9.10		1.13	0.41–3.06		1.21	0.68–2.18		4.9	1.46–16.4		2.1	0.97–4.54	
2nd–3rd T	0.81	0.11–5.87		NA			0.53	0.13–2.15		0.49	0.12–1.97		1.98	0.48–8.21		1.82	0.44–7.53		0.71	0.18–2.90		0.76	0.34–1.72		NA			0.76	0.19–3.07	

NA, not applicable/no child in this category; T, trimester.

**Table 8:** Description of births defects among 13,124 live births (French Perinatal Cohort [ANRS CO1/CO11]).

Organ System Classification (EUROCAT)	*N*
**Nervous system**	60
Spina bifida	3
Hydrocephalus, dilatation of ventricular system	31
Microcephaly	13
Agenesis/malformation of corpus callosum	4
Pachygyria	1
Cerebral cyst, single or multiple	7
Malformation of spinal cord	1
**Eye, ear, face, and neck**	25
Anopthalmos	1
Congenital cataract	1
Congenital glaucoma/buphthalmos	5
Congenital ptosis/malformation of eyelid	8
Malformation of lacrymal duct/apparatus	2
Coloboma of iris	1
Aniridia	1
Congenital corneal opacity/malformation of cornea	4
Absence of auditory canal	1
Cyst of tongue	1
**CHDs**	182
Ventricular septal defects	101
Atrial septal defects	30
Atrioventricular septal defects	1
Tetralogy of Fallot	1
Pulmonary valve stenosis/dysplasia	14
Hypoplastic left heart	1
Coarctation of aorta	6
Tricuspid valve malformation	3
Aortic valve stenosis/malformation	1
Mitral insufficiency	1
Other CHD	2
Patent ductus arteriosus as only CHD in term infants (≥37 wk)	15
Pulmonary artery stenosis/other malformation	6
**Respiratory**	6
Choanal atresia	3
Underdevelopment of nose	1
Congenital cystic lung	1
Hypoplasia of lung	1
**Oro-facial clefts**	9
**Other malformations**	25
**Genetic syndromes and microdeletions**	7
**Chromosomal**	24
**Teratogenic syndromes with malformations**	11
Fetal alcohol syndrome	6
Cytomegalovirus infection resulting in malformation	5
**Digestive**	21
Esophageal atresia	2
Duodenal atresia	1
Imperforate anus	3
Hirschsprung disease	4
Diaphragmatic hernia	3
Fistula of rectum and anus	2
Microcolon	3
Other malformation of intestines	1
Malformation of bile ducts	1
Duplication of digestive organs	1
**Abdominal wall defects**	2
Gastroschisis	1
Omphalocele	1
**Urinary**	53
Renal dysplasia	7
Hydronephrosis/renal pelvis dilatation	25
Renal agenesis, unilateral	5
Renal cyst	2
Pelviureteric junction syndrome	3
Vesicoureteric junction syndrome	2
Megaloureter	3
Duplication of ureter	1
Ectopic kidney	1
Other malformation of kidney or urethra	4
**Genital**	45
Hypospadias	26
Indeterminate sex	1
Ovarian cyst	10
Malformation of clitoris	4
Agenesis of testis, unilateral	1
Micropenis	3
**Limb and musculoskeletal**	133
Talipes equinovarus	14
Hip dislocation and/or dysplasia	25
Polydactyly	61
Syndactyly	2
Reduction defect of upper limb	2
Reduction defect of lower limb	4
Other malformation of upper limb	6
Other malformation of lower limb	2
Arthrogryposis multiplex congenita	1
Achondroplasia, hypochondroplasia	1
Amniotic band syndrome	1
Other malformation of skull and bones	14

Total number of defects exceeds 575 because some children were included in several organ systems. Each child was included only once in each organ system.

**Table 9:** Description of birth defects among terminations of pregnancy (French Perinatal Cohort [ANRS CO1/CO11]).

Organ System Classification (EUROCAT)	*N*
**Nervous system**	5
Anencephaly	1
Spina bifida	1
Agenesis of corpus callosum	1
Anomalies of gyration	1
**CHDs**	4
Severe heart defects	3
Atrioventricular septal defects	1
**Urinary**	1
Renal hypoplasia, bilateral	1
**Other malformations**	5
Multiple congenital malformations	4
Cystic hygroma	1
**Genetic and microdeletion syndromes**	1
Goldenhar syndrome	1
**Chromosomal**	9
Trisomy 21	6
Trisomy 13	2
Unbalanced translocation	1

## Discussion

The prevalence of birth defects in our study population was 4.4% as assessed using the EUROCAT guidelines and 7% using the MACDP classification. This is higher than the rates reported in most large studies including patients exposed to ARV drugs, which were 1.5% in the European Collaborative Study, published in 2005 and thus including different drugs than those used in more recent studies [18], 2.8% in a recent study in the United Kingdom [17], and 2.9% in the APR [13]. The prevalence in our cohort is, however, consistent with that observed in smaller prospective studies, which all report higher rates, from 5.3% to 8.3% [11],[12],[16],[27]. We hypothesize that studies involving few centers may have a higher level of reporting because of greater motivation of clinicians for the specific research program and more intensive data monitoring than in large multicenter cohort studies and registries. In this sense, the higher prevalence in EPF despite the large number of pregnancies and study sites may result from the high level of completeness of our data collection, which is sustained through regular monitoring at study sites and motivated clinicians uniting in an active national network, but may also result from easier referral for free further investigations, facilitated by the French health insurance system.

An important result is that no association was found between birth defects and lopinavir or ritonavir with a power >80% for an OR of 1.5, and for tenofovir, nevirapine, and abacavir with a power >70%. For all these drugs, the power was >95% for an OR of 2. This result is very reassuring in view of the fact that several of these ARV drugs are currently being increasingly used.

We found a specific association between exposure to zidovudine during the first trimester and CHDs, for both classifications. It persisted after adjusting for potential confounding variables, including other ARV drugs, and in all sensitivity analyses, including adjustment for year of birth. The association with heart defects was of a larger magnitude than that with birth defects overall, and no association was found between zidovudine and other types of defects, suggesting a specific association. We found the same risk factors (maternal age and prematurity) for heart defects as in general population [28],[29], which argues against uncontrolled selection biases. The other risk factors were maternal CD4 count <200 cells/ml and female gender. The association between immune status and heart defects may be due to a direct effect of immune status or to cotrimoxazole, which is recommended in patients with CD4 <200 cells/ml [30] but which has been shown to be associated with birth defects [31]–[34]. Unfortunately, concomitant medications were not documented in our cohort. Brogly et al. reported more heart defects for children exposed to zidovudine during the first trimester [11] but stressed that this association was based on a small number of defects, and needed further confirmation. Watts et al. described an association between ART and CHDs, but could not incriminate any drug in particular [14]. Finally, zidovudine was found to be associated with heart dysfunction [35]: this association was stronger for females than for males. Similarly, a gender difference regarding heart dysfunction and zidovudine has been described in an animal study [36]. The potential pathological mechanism has yet to be elucidated. Other studies on birth defects did not find the same association, but most of them lacked statistical power since the numbers of children included were between 344 [37] and 8,242 [17]. The APR, the only previous study with a number of patients similar to that of our study, was not designed as a cohort study and includes data collected from different countries with highly diverse follow-up protocols, which may lead to classification and selection biases [13]. The strength of our prospective cohort in this respect is the free access for all pregnant women in France to standardized prenatal and postnatal evaluations, according to national guidelines, including detailed fetal ultrasound in each of the three trimesters of pregnancy, and meticulous examination at birth and at follow-up visits through the age of 2 y by a pediatrician. We are aware that the prevalence of CHDs may be overestimated in our cohort compared to population studies [29],[38], as they were adjudicated by general pediatricians and not heart defect specialists, as done in some other HIV studies [13],[17]. However this possible overestimation should not lead to differential misclassification bias in the association between these defects and zidovudine, since there was no alert for such risk at the time of the study. The adjudication method was homogeneous throughout our cohort, and, in particular, echocardiograms were performed in children only if there was an anomaly during routine prenatal ultrasound screening or if clinical symptoms such as a heart murmur were present during routine clinical examination. Thus, there was no guideline to perform cardiac evaluations specifically in infants exposed to zidovudine. According to new World Health Organization (WHO) recommendations [39] and current practices in European countries, exposure to zidovudine during pregnancy should decrease in the future, but it remains important to continue investigation into the effects of this drug to evaluate potential consequences for development for the large number of children exposed in the past to this drug.

We found a significant association between exposure to efavirenz during the first trimester and neurological defects, using the MACDP classification: AOR = 3.0 (95% CI 1.1–8.5). This association did not reach significance using the EUROCAT classification (AOR = 2.5 [95% CI 0.9–6.8], *p = *0.13) because this classification excluded a case with a subependymal cyst. The neurological defect rate among children exposed to efavirenz in the first trimester was 1.1%. There has been concern about a teratogenic effect because a preclinical study found three birth defects among 20 monkeys exposed to efavirenz [9], and two cases of neural tube defects were reported in humans [40],[41]. Recently, two studies found an association between efavirenz and birth defects in general [11],[12], with a high prevalence of birth defects (12.8% and 15.6%) among children exposed to efavirenz in the first trimester (47 and 32 children, respectively, for [11] and [12]). The updated APR reported a prevalence of 2.3% among 766 children exposed during the first trimester, not different from children exposed later (1.9% for 160 children). A meta-analysis including these studies found a relative risk of 0.85 [95% CI 0.61–1.20] among 1,437 women exposed to efavirenz in the first trimester compared to non-efavirenz-based regimens [10]. There are two difficulties in studying this association in high-income countries, where detailed studies of birth defects are feasible. First, because the use of efavirenz was, until recently, discouraged in pregnant women and women planning to become pregnant, the number of exposed fetuses is relatively small, and power is lacking. With 372 children exposed to efavirenz in the first trimester, our study is larger than any prospective cohort studies to date. Second, detailed ultrasound examination is recommended, which presumably would lead to the detection of major birth defects early in pregnancy, thus allowing for TOP before inclusion in perinatal studies. The possible non-inclusion of patients with fetuses with major defects because of early TOPs may explain why the association was lower, using the EUROCAT classification, which includes only major defects. Efavirenz is efficacious and inexpensive and is consequently widely prescribed worldwide. Recent guidelines from the US Department of Health and the WHO state that efavirenz does not necessarily need to be changed at the beginning of pregnancy [39],[42]. Our findings are less reassuring, but no causal association can be concluded, because of the small number of defects, and especially since the different neurological anomalies reported do not correspond to a specific malformative pathway.

Didanosine was associated with head and neck defects, whatever the classification. A higher risk of birth defects for children exposed to didanosine has also been reported in the APR, but not in other studies [13]. Our study has the greatest number of children exposed to didanosine during the first trimester, and thus the discrepancy between our results that those of other studies could be due to lack of power in the other studies.

Nevertheless, didanosine is no longer recommended and is not commonly prescribed during pregnancy (6.9% over the last 5 y in EPF).

Finally, a significant association was found between indinavir and head and neck defects. Birth defects associated with PIs have been less studied, because the placental transfer is low. Nevertheless, an increased risk of birth defects was reported in rats exposed to indinavir [43]. As is the case for didanosine, indinavir is no longer prescribed (less than 3% in the last 5 y). For both of these drugs, the absolute numbers of defects in the exposed groups were low, leading to large confidence intervals, reinforcing cautiousness in any causal conclusion.

There is no nationwide description of the birth defect rate in the French general population, but there are exhaustive regional registries. The birth defect rate observed in our study was higher than in the Paris Birth Defect Registry [44]: 4.4% versus 2.3% (*p<*0.001) among live births, with the same classification (EUROCAT) and during the same period. However, interpretation of this difference is difficult and cannot be attributed solely to treatments and/or HIV infection, because of the longer follow-up of children in our cohort and because of potential confounding factors and regional disparities. The rate of birth defects in our cohort changed over time, with an increase between 1994 and 1996, and a slight decrease afterwards. This decrease is unlikely to be due solely to changes in the general population, since the trend in the birth defect rate among live births in the Paris registry was a regular decrease from 2.4% in 1994–1996 to 2.1% in 2005–2009. We chose not to include time period in the main analysis because of collinearity with PMTCT strategies. However, when included in sensitivity analyses, adjustment for year of birth did not change the association between zidovudine and heart defects.

### Strengths and Limitations of the Study

Our study presents many strengths. To our knowledge, it is the largest national cohort of live births among HIV-infected women, with homogeneous prenatal and postnatal follow-up via standardized questionnaires, and thus has higher power than most studies to date, which is reassuring for drugs found to be not associated with birth defects. Women were included prospectively during pregnancy, before any detection of birth defects. Clinical examinations of children were performed during the first 2 y of life, such that it is unlikely that many birth defects were missed. We used the EUROCAT classification, which excludes most minor anomalies and contains very few decision trees, minimizing the need for additional details for classification, which seems to make it more reproducible. We also used the APR-modified MACDP classification in secondary analyses to facilitate comparisons with previous studies. We studied birth defects by organ system, which is a more specific approach than considering the overall incidence of birth defects. Indeed, teratogenic agents generally affect particular developmental pathways and not several pathways randomly. Therefore, although the number of defects per organ system is lower than the total number of defects, the power for finding an association can be higher. Finally, many characteristics of the mother and neonate were available for adjustment for potential confounders.

Our study also presents some limitations. Gestational age at inclusion varied over time, and early TOPs were not captured before 2005. We then decided to exclude stillbirths and TOPs from the main analysis. The reasons were to avoid false associations of major defects with drugs prescribed more often in recent years, and to limit underestimation of major defects likely to be detected earlier in pregnancy for efavirenz than for other drugs. In sensitivity analyses including TOPs and stillbirths, as expected, the association remained unchanged for zidovudine with CHDs, but decreased for efavirenz with neurological defects. We also excluded the small group of women not treated with ART, as relevant variables to adjust for this very precarious situation were not available in the EPF study. Another concern is the risk of false positive associations due to multiple testing. We chose not to perform adjustments for multiple testing because such an approach is questionable for exploratory studies such as this from a methodological standpoint [45]. Additionally, most statistical tests were not significant, and for significant associations, our discussion was based both on (1) hypotheses emanating from previous findings in the literature and (2) the robustness of the findings of the current study, which was important for the association between zidovudine and heart defects. Lastly, we were cautious in any causal interpretation. Except for the interaction of zidovudine with other drugs, we did not address other possible interactions because of the number of possible combinations, and hence potential interaction effects, and the lack of previous literature to guide the analyses. Missing data were excluded as they were rare for most maternal covariates, except for viral load and CD4 cell count, which were not measured routinely before 1996. These covariates were not likely to be related to birth defect detection and reporting.

Another limitation, common to most studies of birth defects and ART, is the lack of data on concomitant medication and alcohol and tobacco use, which in the case of the current study were not available for the whole study period. Cotrimoxazole use was indirectly accounted for by adjusting for CD4 cell counts in sensitivity analyses. Exposure to tobacco and alcohol during pregnancy was reported by 5% and 1.9% of women, respectively, between 2005 and 2010. Whatever their impact on birth defects, these factors were unlikely to be related to the prescription of any particular ARV drug, and thus should not bias the results.

### Conclusions

In conclusion, we found a higher rate of CHDs in children exposed in utero to zidovudine, which should be taken into consideration, given the large number of children exposed to perinatal zidovudine in the world. Potential mechanisms underlying this association must be investigated. This alert reinforces recent recommendations for PMTCT, which no longer consider zidovudine to be the first-line ART during pregnancy [39]. Though the higher rate of neurological birth defects observed in infants exposed to efavirenz in the first trimester must be interpreted with caution, as discussed previously, our results reinforce the importance of careful clinical follow-up of children in case of perinatal exposure to efavirenz, as recommended in the WHO guidelines [39]. In our study, ventricular dilatation, partial agenesis of the corpus callosum with an interhemispheric cyst, pachygyria, and subependymal cyst, detected by routine prenatal ultrasound screening and/or clinical postnatal follow-up, may have been missed by neonatal examination only. The absence of association between birth defects and several ARV drugs that are increasingly prescribed during pregnancy, such as tenofovir, which is the first-line WHO recommendation for PMTCT, is reassuring and may encourage us to explore various zidovudine-sparing regimens. Nonetheless, whatever the impact some ARV drugs may have on birth defects, it is largely surpassed by the major role of ART in successful PMTCT, leading to the decrease of transmission rates from 20% without ART to the current rate of less than 1%.

## Abbreviations

ANRS: Agence Nationale de Recherche sur le Sida et les Hepatites
AOR: adjusted odds ratio
APR: Antiretroviral Pregnancy Registry
ART: antiretroviral therapy
ARV: antiretroviral
CHD: congenital heart defect
EPF: French Perinatal Cohort
EUROCAT: European Surveillance of Congenital Anomalies
IDU: intravenous drug use
MACDP: Metropolitan Atlanta Congenital Defects Program
NNRTI: non-nucleoside reverse transcriptase inhibitor
NRTI: nucleoside reverse transcriptase inhibitor
OR: odds ratio
PI: protease inhibitor
PMTCT: prevention of mother-to-child transmission
TOP: termination of pregnancy
WHO: World Health Organization
